# Quality and Shelf Life of Fresh Meat from Iberian Pigs as Affected by a New Form of Presentation of Oleic Acid and an Organic-Acid Mix in the Diet

**DOI:** 10.3390/foods10050985

**Published:** 2021-04-30

**Authors:** Ceferina Vieira, Ainhoa Sarmiento-García, Juan-José García, Begoña Rubio, Beatriz Martínez

**Affiliations:** 1 Estación Tecnológica de la Carne, Instituto Tecnológico Agrario de Castilla y León (ITACyL), Calle Filiberto Villalobos, 5, 37770 Guijuelo, Spain; VieAllCe@itacyl.es (C.V.); gargarjj@itacyl.es (J.-J.G.); rubherbe@itacyl.es (B.R.); mardombe@itacyl.es (B.M.); 2 Área de Producción Animal, Departamento de Construcción y Agronomía, Facultad de Agricultura y Ciencias Ambientales, Universidad de Salamanca, Av. de Filiberto Villalobos 119, 37007 Salamanca, Spain

**Keywords:** Iberian pig, oleic acid, diet, chemical composition, lipid profile, natural organic-acid mix

## Abstract

The objective of the study was to evaluate the inclusion of a novel form of oleic acid and an organic-acid mix in the diet of Iberian pigs, and their effect on the quality and shelf-life of the pig meat. 200 castrated male Iberian pigs were randomly assigned to four groups. Diets included different fat sources: pig fat (G1), solid oleic acid (G2), oleic-high sunflower oil with solid oleic (G3); a diet of G3 supplemented with organic-acid mix (G4). Pigs were slaughtered at 182 days. Back fat and the longissimus thoracis et lumborum muscles were removed, and nutritive and sensory quality were analyzed. The shelf-life of meat packaged (70%O_2_/30CO_2_) during retail storage up to 21 days were studied. A higher percentage of oleic acid and MUFA, and lower SFA were observed in subcutaneous fat on a G2 diet. G2 resulted in the highest muscle fat content, and G4, the highest cooking losses. In sensory analysis, marbling, tenderness and chewiness were higher in the G2 samples. No differences were found in the bacterial count and sensorial analysis depending on storage time at the end of the experiment, but a lower total viable count was showed at the start of the study in the G4 samples. These results suggest a better assimilation of solid oleic acid. However, the organic-acid mix requires further studies.

## 1. Introduction

The Iberian pig is characterized by the high quality of its meat and its fat content. It is an indigenous breed of the Iberian Peninsula [1]. The Iberian pig has a slow growth rate, so to reduce costs it is common to use a crossbreed with Duroc heritage [2]. Concerning meat production systems, the most widely renowned is the free-range system of breeding in dehesas, with access to the consumption of acorns. The resulting products are destined for a niche market of highly priced dry-cured processed meat [1]. Nevertheless, the compositions of these animal fats can be modified by the nature of the feeding fat, which can affect their metabolic pathways in different ways [3].

Traditionally, the cured products of the Iberian pig are the most valued by international markets. Thus, most studies about the effect of the feeding of Iberian pigs on meat quality have focused on dry-cured products from prime cuts, such as ham, shoulder and loin, or as cured sausages, such as chorizo. However, the consumption of fresh meat has experienced an increase of 35% in the last 5 years [4]. The high quality of fresh meat from Iberian pigs has been demonstrated in various studies [5]. According to previous studies, fat content (marbling) or juiciness are quality traits that are highly appreciated by the consumer [6]. Moreover, the quality and consumer acceptability of meat products from Iberian pigs are related to their high intramuscular fat and oleic acid contents. In addition, due to changes in consumer habits, certain strategies have been applied to keep meat quality and extend the shelf life. For this reason, modified atmosphere packaging has become a common technology for meat preservation, which involves the substitution of air with a gas mixture that serves to retard meat spoilage. The stability of the appealing, bright red color of meat is conditioned by the oxygen pressure, thus gas mixtures containing high oxygen percentages are commonly used in modified atmosphere packaging. Regarding microbial growth, the presence of carbon dioxide reduces the growth of spoilage bacteria and delays meat deterioration. Consequently, to satisfy consumer demand for fresh meat, Iberian pig feeding strategies must be aimed at increasing the nutritional and sensory quality as well as convenience, delaying microbial and sensory deterioration during storage, thereby extending the shelf life.

The digestive characteristics of the gastrointestinal tract of pigs allow the alteration of the fatty acid composition of fat deposits by modifying the fat content in the diet [7]. The lipid composition of pork could be influenced by several factors, but among those, the breed and the type of diet ingested by the pig at the end of the fattening period are the main factors [8,9]. The improved nutritional and sensory traits are generally attributed to the high oleic acid content of the pig fat and to the presence of certain unsaponifiable components associated with the feeding regime [7]. In this regard, the traditional method of raising Iberian pigs fed an acorn-based diet gives around 55% of oleic acid content, and relatively low concentrations of linoleic and palmitic acids (around 8% and 20%, respectively) which provides fat in demand by consumers [10]. Studies have been conducted to find alternatives to the use of acorns. The use of fat-enriched diets is common in the Iberian pig-farming sector. In accordance with this, previous studies have evaluated how the lipid deposits of pigs are modified as a function of the proportion of oleic acid in the diet [11]. The inclusion of high oleic acid sunflower oil (among other monounsaturated fatty acids (MUFA)) in the feed is being used to modify the profile of fatty acids characteristic of pigs fattened using the traditional system based on acorns from evergreen oaks and pasture [12,13]. However, the compositions of these animal fats (i.e., pork tissue fat) can be modified by the nature of the feeding fat, which can affect their metabolic pathways in different ways [3]. As shown, different authors have studied the effect of different sources of fat on the diet; however, to our knowledge, the inclusion of oleic acid in solid form when fattening Iberian pigs without grazing has not been evaluated.

As has been highlighted above, for consumers making a purchase decision, the shelf life is as important as the nutritional value. Thus, producers have implemented feeding to improve the shelf-life of their products. There are several treatments in the prevention of food spoilage, including animal-derived systems, plant-derived products, and microbial metabolites [14]. Among these, organic acids have been utilized as food additives and preservatives for preventing food deterioration and extending the shelf life of perishable food ingredients, because they inhibit the growth of most microorganisms [14,15]. Organic acids are usually the fermentative by-product of ubiquitous organisms such as the lactic acid bacteria (LAB) which produce a major antimicrobial and bio-preservative substance, lactic acid. Some of the organic acids (lactic, acetic, propionic, citric, and benzoic acid) have been used as food preservatives, and these studies have shown antimicrobial activity against the microflora of meat. Previous studies have evaluated the effect of the inclusion of organic acids in food [16,17,18,19]. For example, Bacon et al. [19] reported reductions in *E. coli* O157:H7 levels following the use of a lactic or acetic acid solution rinsing process in beef carcasses. Among all the organic acids that have been tested in previous research, acetic and lactic acids have been the most widely accepted as carcass decontamination rinses [14]. Otherwise, in animal production, most organic acid compounds are used as direct additives in animal feed as antimicrobials [15]. To our knowledge, there is no information available that has analyzed the effect of organic acid consumption in animals on the shelf-life of meat.

The objective of the present study was to evaluate the effect of diets with a novel form of inclusion of oleic acid and a natural compound with a potential organic-acid mix effect on the chemical composition, sensory quality, and shelf-life of Iberian pork meat.

## 2. Materials and Methods

### 2.1. Ethics

As the study was carried out on farm animals, no special certification was required for the breeding of laboratory animals. All procedures followed the European policy for the protection of animals used in research and other scientific purposes [20]. During the development of the experimental test, the specifications included in Regulation (EC) No 2017/893 [21] regarding the controls and other official activities were carried out to guarantee the application of the legislation on food and feed, and the regulations were followed at all times, as were the rules on animal health and welfare, plant health and phytosanitary products.

### 2.2. Animal Rearing and Batches

A total of 200 cross-bred Iberian (Duroc × Iberian) neutered male pigs (*Sus scrofa*) were used in this experiment. The farm is located in Villar de Gallimazo (Salamanca, Spain) (40°58′00.9″ N 5°17′45.9″ W). The initial average weight of animals was 36.7 ± 0.9 kg, and the pigs were randomly distributed into four groups (50 pigs per group). The pigs were distributed across 4 independent areas that had the same environmental conditions. The trial lasted 182 days.

The stocking density was 2 m^2^ of available floor per animal. The floor was partly concrete-slatted and partly fully solid, with fresh straw added every day to cover most of the fully solid area. The animals took the feed from automatic feeders, and water from the bowl drinker (ad libitum).

All animals received three different diets during the experimental period, according to the rearing phase: growing 1 (25–60 kg) growing 2 (60–100 kg), and finishing (100 kg–150 kg). Growing 1 was common for all groups (metabolizable energy = 3195 kcal/kg; crude protein = 15.50%, crude fat = 3.44%), including pig fat as a fat source. The following phases (growing 2 and finishing) were different depending on the treatment (G1, G2, G3, and G4). G1 was the control group, and pig fat was the only fat source in their feed mixture. In G2 to G4 feed mixtures, pig fat was replaced by different fats. In G2, pig fat was replaced with solid oleic acid; in G3, pig fat was replaced with oleic high sunflower oil and solid oleic acid; and G4 received the same diet as G3, supplemented with a natural organic-acid mix. The amount of these products that was included in the feed was in order to obtain 50% of oleic acid with respect to total fatty acids. The pig fat was obtained from an Iberian pig slaughterhouse (Guijuelo, Salamanca, Spain). Solid oleic acid, high oleic sunflower oil, and natural organic-acid mix were provided by a company in the animal nutrition sector (Arganda del Rey, Madrid, Spain). Solid oleic acid is a new form of oleic acid. This ingredient has not been used previously for animal nutrition. This product is made from an olive pomace fatty acid distillate, lime hydroxide and antioxidants. These products were weighed and mixed under conditions that allowed them to react and form calcium salts of these fatty acids. Their characteristics and the rest of the fat sources included in the diet are shown in Table 1. The organic-acid mix included formic acid (8.50%), lactic acid (4%), propionic acid (15%), ammonium propionate (15%) and copper cation (0.25%). Details of the rest of the composition of this natural organic-acid mix are the property of the company that supplied it.

The chemical composition of the experimental diets was analyzed (AOAC, 2003). The fatty acid profile of fat sources of the different experimental diets (G1, G2, G3, G4) was determined by gas chromatography according to the method proposed by Lurueña-Martinez et al. [22]. For amino acid content analysis, the samples were pretreated by acid hydrolysis with hydrochloric acid, and subsequent amino acids were separated by liquid chromatography mass spectometry(LC-MS) (Single Quadrupole LC/MS, Agilent Technologies, Stockport, UK). The ingredient and nutrient content of the different treatments are shown in Table 2. All diets at the same rearing phase (growing 2 and finishing) were isoenergetic and isonitrogenous, and provided all the requirements of swine.

### 2.3. Carcass Sampling

After 182 days, pigs were transported to the local slaughterhouse (Guijuelo, Salamanca, Spain) and were kept in lairage for 12 h with full access to water but not to food, according to official Spanish standards. Previous to slaughter, the pigs were weighed individually. Pigs with a mean live weight of 156.8 ± 3.3 kg were slaughtered. Animals were slaughtered by electrical stunning and exsanguination. From 50 animals slaughtered per treatment, 10 carcasses of each were sampled.

#### 2.3.1. Subcutaneous Back-Fat Sampling

The back-fat thickness was measured at the level of the 3rd lumbar vertebra and 6th rib using a digital caliper for all pigs. To determine the fatty acid profile, a sample of adipose tissue (approximately 5 × 5 cm^2^) was extracted from each animal. The tissue comes from the back fat at the level of the last lumbar vertebra. The samples were vacuum-packed and kept at −20 °C until analysis.

#### 2.3.2. Meat Sampling

The *M. longissimus thoracis et lumborum* muscle of the left carcass side was removed. The samples were transported under refrigeration (4 ± 1 °C) to the Estación Tecnológica de la Carne (Guijuelo, Salamanca, Spain) for the corresponding analyses. After arrival at the laboratory, the longissimus muscle was divided into subsamples as follows. The longissimus thoracis between the 6th and 8th rib sections were used for chemical analysis, and were vacuum-packed and kept frozen at −18 °C. The pH and color measurements were recorded in the longissimus surface at the 8th rib level, and steaks of about 4 cm were also vacuum-packed for testing cooking losses and texture analysis. The remaining portions of longissimus thoracis, about 6 cm-thick, were also vacuum-packed and kept frozen for sensory analysis. The *longissimus lumborum* was used for shelf-life analysis.

### 2.4. Fat and Meat Characteristics Analysis

#### 2.4.1. pH and Instrumental Color

The pH and color were measured on the *m*. *longissimus thoracis et lumborum* muscle at the 8th rib level. The pH was measured with a Crison pH Meter BASIC 20^®^ (Hach Lange Spain, L’Hospitalet de Llobregat, Barcelona, Spain) equipped with a penetration electrode. For instrumental color measurement, the muscle surface was allowed to bloom at room temperature for 90 min after cutting. Instrumental color evaluation was made throughout using colorimetric parameters (L* (lightness), a* (redness), b* (yellowness), H* (hue) and C* (saturation) (CIE, 1976)). Measurements were recorded using a Minolta 2006d spectrophotometer in the CIE L* a* b* space under D65, 10°, and SCI conditions.

#### 2.4.2. Proximate Analysis

A NIRS system method was used to analyze the chemical meat composition. A representative sample of LTL muscle was ground up and placed in the cups of the equipment. Spectra were recorded at 32 scans (400–2500 nm) per sample, using a benchtop XDS NIR Rapid Content Analyzer, and data were analyzed by the software installed on the equipment.

#### 2.4.3. Determination of the Fatty Acid Profile

For the determination of the fatty acid profile of subcutaneous fat and intramuscular fat of the *longissimus thoracis* muscle, total lipids were extracted according to the method described by Folch et al. [23]. Methyl esters were then obtained according to the method described by Lurueña-Martinez et al. [22]. 4 mL of hexane and 200 µL of KOH 2M, in anhydrous methanol, were mixed with subcutaneous fat or intramuscular fat, respectively. The mix was shaken (30 s at 2000 rpm approx.) and after centrifugation, the organic phase was collected in vials. Two µL was injected into an Agilent Technologies 6890 gas chromatograph (GC) (Santa Clara, CA, USA) using a 100 m × 0.25 mm × 0.20 μm fused silica capillary column (SP-2560, Supelco, Inc, Bellefonte, PA, USA), which was equipped with a split/splitless injector and a flame ionization detector (FID). Fatty acid methyl esters (FAMEs) were separated on a 60 mm DB-23 fused silica column with an i.d. of 0.32 mm and a 0.25 µm film thickness. The temperature program to determinate subcutaneous fatty acid profile was as follows: the initial temperature was 190 °C (2-min hold), followed by an increase from 190 to 230 °C (5 °C/min). The GC parameters were as follows: injector temperature 260 °C, detector temperature 260 °C, and the carrier gas was nitrogen at 1.0 mL min^−1^ and split ratio 1:50. However, to determine the fatty acid profile of intramuscular fat, the oven temperature was 200 °C for 60 min. The temperature of the injector and detector was 260 °C. The carrier gas was helium at 1 mL/min and the split ratio was 1:20. In both cases, the fatty acid contents were calculated using chromatogram peak areas and were expressed as g per 100 g of total fatty acid methyl esters. In addition, the percentage of saturated fatty acids (SFA), monounsaturated fatty acids (MUFA), polyunsaturated fatty acids (PUFA) and the ratio *n-6*/*n-3* were calculated.

#### 2.4.4. Cooking Losses and Texture Analysis

To determine the weight loss after cooking, the method was followed as proposed by Honikel [24]. Samples were packaged in plastic bags and heated to 70 °C until they reached an internal temperature of 70 °C. Before removing the samples from the heat, it was verified that the interior of the sample had reached 70 °C by using a digital thermometer (Hanna Instruments, Woonsocket, RI, US). Once this temperature was reached, the samples were placed in a container with cold water for 30 min. Subsequently, the samples were dried. The samples were weighed before and after cooking, and losses were calculated using the following equation: % weight loss = ((W0−Wf)/W0) × 100, where W0 is the weight before cooking and Wf the weight after cooking.

Samples from cooking loss analysis were subjected to the Warner-Bratzler shear force test [23] using the Texture Analyzer TAXT2i (Stable Micro Systems, Surrey, UK) equipped with a Warner-Bratzler device. A minimum of 8 strips, 1 cm × 1 cm cross-section and fiber parallel to the longest dimension of at least 2 cm so that the fiber axis was perpendicular to the Warner-Braztler device, were obtained from each sample. The crosshead was mm s^−1^. The shear force parameter was obtained, and the value showed was the average of all strips evaluated.

#### 2.4.5. Sensory Analysis

Eight trained sensory judges (according to the International Standard method UNE-ISO 13300-2, 2014) performed the sensory assessment.

Previous to the sensory analysis, samples were frozen vacuum-packed and thawed at 4 °C for 24 h; the meat was sliced, taking into account that the sensory analysis is performed in fresh as well as in cooked meat. For fresh meat evaluation, the steaks remaining uncooked were cut into 2 × 2 cm^2^ cubes. For cooked meat evaluation, 2 cm-thick chops were placed between aluminum foil and cooked between the two plates of a grill (Clajosa^®^, Barcelona, Spain), previously preheated to 220 °C. The internal temperature was controlled with a temperature probe, considering that the samples would be cooked when they reached an internal temperature of 70 °C. Each cooked steak was cut into 2 × 2 cm^2^ samples, wrapped in aluminum foil and codified. The different analyzes were carried out in standardized tasting booths, in accordance with the UNE-EN ISO 8589: 2010. To keep the cooked samples warm until their analysis, they were placed in sand baths (212, Solac^®^, Alava, Spain) located in the tasting cabins and heated to 60 °C. Mineral water and unsalted bread were provided in all the booths, to eliminate residual mouthfeel after each sample was consumed.

Since the objective of the study was to characterize the meat of the experimental groups, a descriptive profile was carried out. Different attributes were assessed using a structured linear scale of 5, where the lowest value (1) corresponded to the lowest intensity of each parameter and the highest value (5) to the highest intensity of the same. The analysis comprised two parts. Firstly, the fresh external appearance was evaluated: fresh muscle color, marbling grade and fat distribution, and fat color. In cooked samples, panelists assessed the samples in terms of color, odor intensity, tenderness, chewiness, juiciness, and quality and intensity of flavor.

### 2.5. Shelf Life of Meat

#### 2.5.1. Sample Preparation

*Longissimus thoracic et lumborum* muscles were sliced in chops (about 2 cm thick) that were placed randomly in trays corresponding to the sampling times established during storage (D0, D4, D8, D11, D16 and D21 days). Since microbial analysis is destructive, a tray for carrying out microbial analyses and a tray to perform the sensory and physicochemical evaluation were prepared. The gas mixture flushed into the trays was 70:30%/O_2_:CO_2_ and were closed by heat-sealing (TECNOTRIP mod. TSB-100) with the film (55 μm thick, O_2_ permeability 5 cc/m^2^/24 h/bar at 23 °C/50% RH and 19 g/m^2^/24 h at 23 °C/90% RH of steam permeability). The pool of trays was placed randomly in a refrigerated display case with white fluorescent light (620 lux) at 4 ± 1 °C. The display case was set to 12-h light-dark cycles as in a retail point of sale. In order to minimize the possible differences in light intensity, trays were rotated daily. Each sampling day, the assigned trays were removed from the cabinet to be analyzed.

#### 2.5.2. Gas Composition and Microbiological Analysis

Each sampling day, the gas composition was measured with a portable O_2_/CO_2_ analyzer (Oxybaby, Carburos Metónicos, SA, Madrid, Spain).

Microbiological analysis was conducted as follows. Just after the pack was opened, 10 g of sample were placed in a stomacher bag with 90 mL of sterile peptone water (Scharlau, Barcelona, Spain) to obtain the 1:10 dilution. The sample was homogenized with a laboratory blender (PK 400 Masticator, IUL S.A., Barcelona, Spain) for 2 min, and then serial decimal dilutions were prepared in sterile peptone water. In duplicate, 1 mL or 0.1 mL samples of appropriate dilutions were placed onto selective agar plates. Total viable counts (TVC) were determined on a 3M Petrifilm Aerobic Count Plate which was incubated at 30 °C for 72 h. *Enterobacteria* were incubated on a 3M Petrifilm Enterobacteriaceae Count Plate at 37 °C for 24 h. *Pseudomonas* and *Brochothrix thermosphacta* were incubated on Pseudomonas Agar, with Cetrimide Fucidin and Cephaloridine supplement and streptomycin thallous acetate actidione agar (STAA, Oxoid, Spain) with STAA selective supplement, respectively, at 25 °C for 48 h. Lactic acid bacteria (LAB) were incubated on de Man, Rogosa and Sharpe agar at 30 °C for 72 h.

#### 2.5.3. Sensory Evaluation

Sensory analysis was employed to simulate the variables involved in a purchase decision at the point of sale, as when opening the tray at home, thus the analysis was performed with raw meat. On each sampling day, meat packages were assessed for appearance, off-color and off-odor by 8 trained sensory judges (UNE-EN ISO 8586:2014). The appearance was tested in unopened trays, using a 5-point structured scale, from 1 (excellent, fresh meat) to 5 (extremely undesirable). The brownish meat was measured by the percentage of surface discolored (1, none; 2, 1–10%; 3, 11–20%; 4, 21–60%; and 5, 61–100%) was also evaluated in unopened trays, Once the film was removed, panelists evaluated the odor (1, no off-odors; 2, slight off-odors; 3, small off-odors but not spoiled; 4, clearly recognizable off-odors; and 5, extreme off-odors). A mean score of 3 was established as the threshold of consumer acceptability. Shelf life was established as the time to reach a score of 3 [8,12,13].

### 2.6. Statistical Analysis

A one-way ANOVA was carried out to evaluate the effect of the experimental diet (G1, G2, G3 and G4) on meat quality. When statistical differences were detected, the Tukey honest significant difference (HSD) test was used to measure differences between means.

For shelf-life evaluation, data were analyzed using a general linear model (GLM). The model included the effects of diet (G1, G2, G3 and G4) and sampling times during the storage period (0, 4, 8, 11, 16 and 21 days), and the interactions between them. When the interaction was significant, planned comparisons were performed using the Tukey HSD test (comparing diets for each storage period and the storage period for each diet). The statistical differences were defined as *p* < 0.05 and trends as *p* < 10. The statistical package used was Statgraphics Centurion XVII (StatPoint Inc., Warrenton, VA, USA).

## 3. Results

### 3.1. Effect of Diet on the Carcass Yield

The carcass characteristics are shown in Table 3. The weight of the Iberian pigs before slaughtering was 164.6 ± 9.5 kg on average, without significant differences (*p* = 0.671) between experimental groups. No differences (*p* = 0.365) were also found in carcass weight between the experimental groups. Regarding the thickness of back fat, a significant effect was only observed when the thickness was measured at the level of the 3rd lumbar vertebra (*p* = 0.03). The pigs from the control group (G1), which had received pig fat as a fat source, had a higher value of fat thickness than those in G2 and G3. Those in G4 showed intermediate values.

### 3.2. Effect of Diet on Fatty Acid Profile

#### 3.2.1. Fatty Acid Profile of Subcutaneous Fat

The values of the fatty acid profile of the subcutaneous back-fat samples are shown in Table 4. The major fatty acids in subcutaneous fat in all treatments were palmitic acid (C16:0) as saturated fatty acids (SFA); oleic acid (C18:1 *n-9*) as monounsaturated fatty acids (MUFA); and linoleic acid (C18:2 *n-6*) as polyunsaturated fatty acids (PUFA). Regarding the effect of diet on the fatty acid profile, heptadecenoic acid (C17:0) showed significantly higher values (*p* = 0.049) for the G3 and G4 groups. Significantly lower percentages of oleic acid (C18:1 *n-9*) were observed in subcutaneous fat (*p* = 0.013) in G3 and G4 groups with respect to G1 and G2 groups. No significant effect of diet (*p* > 0.05) was observed for any of the rest of the fatty acids studied.

A higher content (*p* = 0.031) of SFA was observed for G3 and a lower value in G1 and G2; G4 showed intermediate values. However, the MUFA content was higher (*p* = 0.026) in G2 than in G3, with groups G1 and G4 had intermediate values. The *n-6*/*n-3* ratio also showed significant differences (*p* = 0.010) between the groups. Group G4 showed a lower value compared to groups G1 and G2, while group G3 had intermediate values.

#### 3.2.2. Fatty Acid Profile of Intramuscular Fat

The fatty acid profile of intramuscular fat of the longissimus thoracis muscle on different experimental diets is shown in Table 5. In this case, the most abundant fatty acids in m. longissimus thoracis et lumborum was palmitic acid (C16:0) as SFA, oleic acid (C18:1 *n-9*c) as MUFA and linoleic acid (C18:2 *n-6*c) as PUFA. However, margaric acid (C17:0) was found in a lower amount with the SFA, while in the case of the MUFA it was myristoleic acid (C14:1), and in the PUFA it was Cis 9,11,13-octadecatrienoic acid (C18:3 *n-4*).

Lauric acid showed lower values (*p* = 0.023) for G1 and higher values with respect to the rest of the groups (G2, G3 and G4). Myristoleic acid showed differences (*p* = 0.000) between groups. Lower values were found in G1 and G2 groups than in the G3 and G4 groups. Conjugated linoleic acid (C18:2 t10-c12) showed a lower value (*p* = 0.074) for the G4 group, with respect to the rest of the groups (G1, G2 and G3). However, arachidonic acid (C20:4 *n-6*) was significantly higher (*p* = 0.088) in the G4 group. For cis-13-docosenoic acid (C22:1 *n-9*) and docosahexaenoic acid (C22:6 *n-3*), two clearly differentiated groups (*p* = 0.004, *p* = 0.026, respectively) were established. Groups G3 and G4 showed a higher content of those acids compared to groups G1 and G2. However, in the case of adrenic acid (C22:4 *n-6*), the G3 and G4 groups had a lower value (*p* = 0.024). The rest of the fatty acid analyses did not show significant differences between experimental groups.

Regarding fatty acid ratios, different fat sources only affected (*p* = 0.000) the ratio *n-6*/*n-3* of the intramuscular fat, with higher proportions in G3 and G4 than in G1 and G2 groups.

### 3.3. Effect of Diet on Meat Quality Traits

The proximal chemical composition and instrumental quality parameters of the longissimus thoracis muscle are shown in Table 6. No differences were found between different groups for moisture and protein, but a significant increase (*p* = 0.047) in fat content was observed for G1 and G2. G4 showed the lowest fat content and G3 showed intermediate values.

Color (L*, a*, b*), pH and texture (WBSF) did not present differences (*p* > 0.05) between the meat of the different dietary treatments. However, statistical differences were found (*p* = 0.003) in cooking loss, higher values of weight loss, and therefore, lower WHC was observed in G4 with respect to G2. G1 and G3 show intermediate values for WHC.

#### Sensory Analysis

Regarding sensory analysis, two groups of parameters were evaluated, the appearance of fresh meat and the sensory characteristics of cooked meat (Table 7). With regard to the external appreciation of fresh meat, no differences were found in fresh color (*p* = 0.925). A lower marbling score (*p* = 0.002) and lower homogeneity of visual fat distribution (*p* = 0.010) were observed in G4. In both parameters, the highest values were found in G2.

For cooked meat, chewiness (*p* = 0.014) and tenderness (*p* = 0.036) showed similar trends. Both showed the highest values for group G1 and G2, with the lowest value being group G4. Group G3 showed intermediate values. The value of juiciness (*p* = 0.002) was lower for groups G3 and G4, while the highest value was shown in group G1. Group G2 had intermediate values. Finally, the overall liking score (*p* = 0.016) was better in group G2 and worse in group G4, while group G1 and G2 showed intermediate values.

### 3.4. Effect of Diet on Shelf Life of Meat Packaged in Modified Atmosphere during Storage

#### Microbiological Analyses

The data on the microbiological analysis of meat from Iberian pigs packaged (70:30%/O_2_:CO_2_) during storage, corresponding to the dietary treatments studied, is shown in Table 8. In general, the microbial populations increased with refrigerated storage time. Because significant interactions were found in some parameters, planned comparisons of dietary treatment and days of storage are shown.

Regarding total viable counts (TVC), between experimental treatments, no relevant differences were observed initially (D0), counts ranging between 3.3 and 4.1 log cfu g^−1^. Until D11, only slight differences (*p* < 0.1) were observed in the TVC, which increased significantly (*p* < 0.05) after D11 in all groups except in G1, which remained stable for up to 16 days of storage (D16). At the end of the experiment (D21), all groups showed values near the limit of 7 log cfu g^−1^ (the limit after which a product is considered no longer suitable for consumption, ICMSF, 1986). *Enterobacteria* counts, considered as a hygiene indicator, remained almost stable from the beginning of the trial to the 11th day of storage, when they began to increase significantly in all the dietary treatments (*p* < 0.05). Only one trend (*p* = 0.086) was observed in *Enterobacteria* counts at the end of the storage, this being that the value of G4 was higher than G2 and G3, with G1 showing intermediate values.

With regard to LAB, facultative anaerobic bacteria with the ability to grow in the presence of CO2 presented a similar behavior to *Enterobacteria*, beginning to increase significantly (*p* < 0.05) from the 11th day onwards, except in the case of G3, when values increased significantly from the 8th day. In all experimental groups, the lactic acid bacteria count continued to increase until the end of storage, when it reached counts ranging between 5.4 and 6.4. It is interesting to note that, although no significant differences were found (*p* = 0.129), the counts of G2 and G4 presented numerical values 1 cfu g^−1^ lower than G2 and 0.5 cfu g^−1^ lower than those of G3. However, no significant differences (*p* > 0.05) were found at any of the sampling points. *Pseudomonas* counts were lower in G2 and G4 from the 8th to the 16th day of storage than in the other treatments. However, no significant differences (*p* > 0.05) were found at the end of the trial. *Brochothrix thermosphacta* counts remained stable throughout all the storage time in the case of G4, while in the rest of the treatments they began to increase after the 11th or 16th days (D11 to D16). As a result, significant differences (*p* < 0.05) were found between dietary treatments on the 16th day, when the G4 and G3 groups showed lower values (*p* < 0.05) than the rest of the treatments. Although G4 presented values 1cfu g^−1^ lower with respect to the other groups, no significant differences were detected (*p* > 0.05), probably because of the high variability of data.

**Figure 1:**
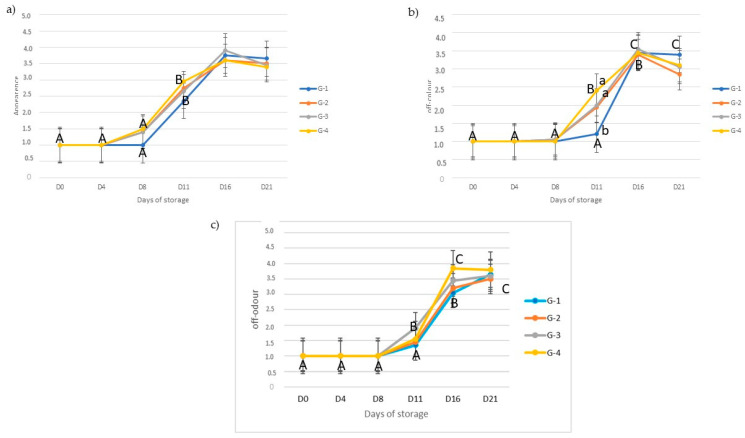
Effect of experimental dietary groups and storage time on the evolution of appearance (**a**), off-color (**b**) and off-odor (**c**) on m. longissimus thoracic et lumborum of Iberian pigs under retail conditions. G1: control (pig fat); G2: solid oleic acid; G3: oleic high sunflower oil + solid oleic acid; and G4: oleic high sunflower oil + solid oleic + natural organic-acid mix. A, B, C averages for each microbial group with a different capital letter mean significant differences (*p* < 0.05) between days of storage within diets. a, b: averages for each microbial group with a different lower-case letter mean significant differences (*p* < 0.05) between diets within days of storage.

Figure 1 shows the scores given by sensory judges to samples packaged under a gas mixture (70:30%/O_2_:CO_2_) at different sampling points during storage. As expected, no differences were detected between treatments (*p* > 0.05) in the freshly cut meat (day 0), when all samples had a score of 1. As was predictable, sensory quality decreased gradually and reflected the decrease of the optimal appearance, color and odor of pork meat during storage. No differences were found between treatments at any sampling point (*p* > 0.05), regarding the appearance of the pork meat during storage. As regards the presence of off-color, evaluated as the percentage of the meat sample surface that was discolored, differences between treatments were detected at D11 (*p* = 0.004), corresponding in G1 to the lowest values. However, all experimental groups showed a similar pattern of a decrease in desirable appearance and the presence of off-color at D16, when all treatments reached scores above 3, which was set as the threshold for refusal. Meat from G3 and G4 presented higher scores of the presence of off-odor (*p* < 0.05) at D16 than those from G1 and G2; between them no differences were found (*p* > 0.05). Thus, the threshold score was just reached at this sampling point in G1 and G2, while G3 and G4 greatly exceeded this threshold.

## 4. Discussion

### 4.1. Carcass Yield and Meat and Fat Composition

Diet has been considered as the main factor that can affect the composition of tissues, and consequently the lipid composition [13]. This fact is significant because it determines the quality of the fat and the meat [3,6], as well as its suitability for processing [5,25]. In this sense, the consumption of acorns and grass from Iberian pigs reared outdoors has been related to the highest-quality meat products [8,9]. The increase in the consumption of Iberian pork products, and the limited production of acorns, have led to the use of concentrates enriched with MUFA, which is becoming a common practice to achieve FA profiles in animal tissues similar to those obtained with the consumption of acorns, as the fatty acid profiles of the animal’s tissues are expected to reflect the fatty acid composition of the feeds received [7,12].

No differences were found, either in the live weight of the animals or in their carcasses, between the different groups studied. There were also no significant differences for the fat thickness at the 6th rib. However, the thickness of back fat at the 3rd lumbar vertebra showed differences between the experimental groups. The average thickness of back fat was slightly lower than that found by previous studies [26,27,28]. The different behavior of subcutaneous fat in different locations of the carcass may be related to the allometric growth coefficients at different anatomical locations [29]. Subcutaneous fatty tissue develops in the distal areas in the early stages of growth, while in the proximal areas it develops to a greater extent in the fattening phase.

Regarding lipid fractions of subcutaneous fat, SFA were lower in G2 and MUFA were higher in G2. For the group on G3, the opposite was observed. This is justified by the variations in oleic acid content that have been observed. In accordance with those results, Ovilo et al. [12] showed variations in the lipid fractions when high oleic sunflower oil was included in the diet. Similarly, those authors showed a greater percentage of MUFA and a lower percentage of SFA in animals that had received high oleic sunflower oil. In our work, no differences were detected in the PUFA content that resulted from the different fat sources in the pigs’ diet. In contrast to our results, Nuernberg et al. [30] showed that feeding olive oil to pigs caused a decrease in PUFA, both in back fat and in total lipid content. In the same line, González et al. [7] only showed an increase in the PUFA content while the rest of the lipid fractions were not affected by the use of two different sources of fat in the diet. In addition, the G4 group showed a decrease in the *n-6*/*n-3* ratio, which has been related to health benefits [31].

The carcass value of the Iberian pig is set in the market according to the major fatty acid proportion in the subcutaneous fat, particularly regarding the C18:1 *n-9* content [32]. A decrease in oleic acid levels (C18:1 *n-9*) in subcutaneous fat was observed for groups G3 and G4, showing the highest value of this acid in group G2. However, the oleic acid content in the feed of group G2 was slightly lower than those of G3 and G4. This fact could indicate better assimilation of the pig’s oleic acid in solid form in a unique way in the diet, compared to the combination of two fat sources (G3) or with organic-acid mix (G4). This could suggest a slower absorption in the gastrointestinal tract of oleic acid in solid form, increasing the incorporation of this acid into the adipose tissue of the animal. Regarding the effect of the format of oleic acid, this could be more similar to pigs fed under free-range conditions than to pigs fed in a feedlot with MUFA-enriched diets. In this sense, [27,28] reported higher levels of C18:1 *n-9* in Iberian pigs fed under free-range conditions than in pigs fed in feedlots with MUFA-enriched diets. In contrast to those results, previous authors [7,9,12,33] did not find differences in C18:1 *n-9* levels when pigs were fed with enriched MUFA. This fact is probably related to the high level of C18:1 *n-9* (68.50%) in the concentrates used in our study than that used in other studies (20% in González et al. [7], 24% in Ruiz et al. [33]). Benitez et al. [34], highlighted that the interconversions between fatty acids limit the impact of dietary additions. However, the total amount of fat is not modified as it depends mainly on the diet [32]. Several works [30,31] reported an exponential increase in C18:1 *n-9* in back fat due to feeding with an oleic acid-rich oil. This fact would consolidate the theory set forth above, referring to greater assimilation of oleic acid in the solid form (G2) as observed in the fatty acid profile of subcutaneous fat. These authors indicate that the direct deposition of fatty acids is carried out through a series of complex mechanisms of digestion, absorption and transport, so that the fatty acids accumulated in this way depend on the composition of the ration. Regarding the effects of the oleic acid format included in the feed on absorption, there is little information on the digestibility of the different fat sources, as well as on their quality; although there is agreement that both metabolic factors impact the animal and make use of the same [34,35]. The PUFA content depends on the characteristics of the fat supplied in the diet (content and structure), de novo synthesis of fatty acids, the interconversion rate to other fatty acids and metabolites and the oxidation reactions for energy consumption. The chemical composition and technological properties of the loin (m. longissimus thoracis et lumborum) were analyzed. No differences were found for moisture and protein depending on the fat source received. Nevertheless, in spite of this equal energy and protein supply, differences were observed in the amount of fat in the muscle. Benítez et al. [34] reported an effect on fat muscle content in spite of the fact that the rations offered to Iberian pigs were isoenergetic isoproteins. However, elevated dietary fat concentration may act as a constraint on feed intake through a reduction in the digesta passage rate. The use of pig fat (G1) and oleic acid in solid form (G2) in the diet of pigs showed an increase in the fat content in the muscle. It seems that the mix of two forms of oleic acid gives rise to an increase in the fat absorbed.

Although the oleic acid content of subcutaneous fat was affected by the use of different forms of fat in the diet, the oleic acid content of the loin did not show differences between the groups. However, Nuernberg et al. [30] studied the effect of olive oil added to the Iberian pigs’ diet and found that it results in a high oleic acid percentage and higher MUFA content in intramuscular fat, with respect to no supplements in the diet. For the *n-6*/*n-3* ratio there was a change in the trend observed in subcutaneous fat. A higher value (*p* = 0.010) of the *n-6*/*n-3* ratio was observed for groups G1 and G2. Previous authors [3] proposed that the effect of fat-enriched diets on the composition of different tissues is unclear, since the results are not consistent. In this sense, the different dietary treatments (high inclusion or moderate inclusion of these fats in an enriched diet) make their comparison difficult regarding tissue (muscle type) and sampling [27]. For example, Daza et al. [32] reported significant effects on subcutaneous back fat and *semimembranosus muscle* but not on the *B. femoris* when pigs received a different percentage of fat in the diet. In addition, the different functions of endogenous synthesis of FA, the direct deposition through diet, and the regulation of FA synthesis by diet ingredients are key factors in pig lipid metabolism and production, thus it is not clear enough [8]. In accordance with these results, those authors reported that adipose tissue reflected the dietary modifications in terms of total tissue FA content to a greater extent, but the effect of experimental treatment on muscle FA was not so evident. In relation to the effects of the oleic acid format included in the feed on absorption, there is little information on the digestibility of the different fat sources, as well as on their quality; although there is agreement that both factors impact the metabolic use that the animal makes of it [35,36]. In any case, the fatty acid composition of loin fat in the groups studied results in a carcass rich in C18:1 *n-9* (>50%) and with a very low concentration of palmitic (C16:0) and stearic (C18:0) acids (below 26% and 13%, respectively). This fact makes this a healthier meat than that which comes from pigs fed in a traditional way, which could increase the consumption of these products [13].

### 4.2. Meat Quality Traits

No differences in color were observed depending on the fat source used. This is in accordance with Serrano et al. [37]. Nevertheless, the color values (in terms of L*, a* and b*) differed slightly from those published by other authors for Iberian pigs [38], showing slightly higher values for L* (34–54) and lower for a * (7.5–14.8). These differences may be justified by the varieties of Iberian pig used, the food received and the housing systems for raising the animals. In comparative studies in which Iberian pigs have been contrasted with breed crosses with Duroc pigs [5], the pigs from the Iberian genotype show redder (higher values of a*) and darker (lower values of L*) muscles and a higher level of intramuscular fat in the longissimus muscle than in the other pig types.

The water and fat content and its distribution also have a major influence on meat properties, especially its tenderness, juiciness and appearance. Thus, WHC is a physico-chemical parameter affected by pH values, composition traits such as different intramuscular fat and moisture contents, muscle fiber characteristics or even the endogenous organic-acid mix or antioxidant content [38,39,40]. Cooking loss values observed in our study are in the range of those reported in studies on Iberian pig meat by Tejerina et al. [5]. Likewise, no differences were observed in pH between the experimental groups, so the differences found in cooking loss should be attributable to chemical muscle composition. In this sense, the G4 group showed higher cooking losses, and thus presented a lower WHC compared to the rest of the groups. Tejerina et al. [5] also found a higher WHC in different muscles of an Iberian pig raised with grazing and feeding on acorns and grass. In our study, the higher percentage of cooking loss showed by G4 could be related to the lower fat content, as is observed in different pig breeds [37,41,42,43].

Both the fat and its consistency are of great importance for Spanish consumers of Iberian pork [1,26]. Oleic acid has a great influence on the consistency of fat [7,13]. Although no differences were found in the oleic acid content of loin fat, differences were shown in the degree of fattening (0.002) and fat distribution (0.010). In both cases, these parameters showed higher values for the group that had only received solid oleic acid as a source of fat in the diet (G2). However, the group that combined the two types of fat source and the natural organic-acid mix (G4) showed the lowest values. These parameters show a positive relationship with the observed fat content (Table 5) and affect sensory parameters. Odor intensity has been related to the amount of SFA [44]. As shown in this study, no differences were found for both parameters. For the chewiness (0.014) and juiciness (0.002), a lower value for G4 was observed than for the rest of the groups. This could be justified by the increase shown in cooking losses and a lower percentage of fat. Juiciness is made up of the combined effects of the initial fluid release and the sustained juiciness resulting from the stimulating effect of fat on salivary flow [45]. The decreases in fat content decrease the sensation of juiciness in the oral cavity after chewing, due to the lubrication of the food [8]. In accordance with those results, Ruiz et al. [46] related an increase in juiciness to an increase in fat and moisture. Although WBSF did not show significant differences between the different groups studied after cooking the meat, the panel of tasters expressed differences between the groups for the tenderness of the meat (*p* = 0.036). Differences between the assessment of firmness by a panel of tasters and the instrumental measure are due to the fact that the taster, when assessing the firmness, involuntarily takes other characteristics of the food into account. An increase in tenderness was observed in the G2 group, which had shown a higher percentage of fat. Intramuscular fats, present in and around the muscle fibers, lubricate the fibers and fibrils and so make for a more tender and juicier product that potentiates the sensation of tenderness. Thus, tenderness is closely associated with juiciness [45,46]. To summarize, scores indicated a better result for G2, followed by G1 and G3, and the worst result was G4 (*p* = 0.016). This fact is justified, as seen above, by a greater degree of fattening, fat distribution and chewiness.

### 4.3. Shelf Life of Meat in Retail Conditions

It is known that packaging influences the extension of shelf life in raw chilled meat by inhibiting or retarding the growth of undesirable microflora. Modified atmosphere packaging (MAP) is used to protect products against deteriorative effects, and has been established as an effective technology, improving the quality and extending shelf life. MAP is the replacement of air with another gas mixture before sealing in barrier materials, with CO_2_, N_2_ and O_2_ gases being the most commonly used. The major cause of spoilage is contamination by microorganisms during processing from animal slaughter to meat products [47,48]. Spoilage depends on the number and type of microorganisms, and microbial proliferation during storage. Besides, the majority of fresh meat MAP has a high O_2_ and this may cause deterioration in quality. There are several deteriorative effects of spoilage in meat, such as discoloration, off-flavor and off-odor development, texture changes, and even nutrient loss. These spoilage defects generally occur through different mechanisms. Deteriorative changes during meat storage can be caused by natural processes in meat, such as biochemical oxidative processes or metabolic reactions from biological membrane disruption in the muscle cells after slaughtering. As expected, an increase in the count of all microorganisms was observed from the beginning of storage (D0) to the end of storage time studied (D21) (*p* = 0.000). Luong et al. [47] have noted that high O_2_ concentrations are useful for red meat color stabilization, while increasing CO_2_ content in MAP is useful for inhibiting microbial growth. Concerning gas composition, as expected, the 70/30% O_2_/CO_2_ atmosphere used in this work changed substantially during storage. As described, the modified atmosphere packaging (MAP) used was 70% O_2_/30% CO_2_. As storage progressed, O_2_ decreased while CO_2_ increased in all trays (data not shown). On average, O_2_ decreased from 68.5% to 69.5% at D0, down to 54.6% to 62.3% at the end of storage. CO_2_ increased from 28.6% to 29.3%, up to 33.1% to 27.4%. These changes in the composition of the tray headspace could be due to the solubilization of CO_2_ in the product. Regarding microbiological quality, as a whole, the initial bacterial charge indicated good hygienic operations in both the slaughter and processing. However, it is interesting to note that slight differences were found at D0, the counts of TVC being higher in G1 and G3 than in G1 and G2. This difference was noted throughout the storage in the same microbial groups and sampling points. Nevertheless, no differences were observed between the different pig groups for any microbial groups at the end of storage. According to Spanish legislation (Regulation EC [49]), the limit established for bacterial counts is 10^6^ CFU/g, and these counts were generally reached at the end of storage both in TVC and LAB. Regarding the evolution of microbial groups during storage, *Enterobacteria* increased significantly from D8 in G1 and G3 or D16 in G2 and G4. Insausti et al. [50] reported that Enterobacteriaceae are one of the main microorganisms that caused spoilage of refrigerated meats packaged under modified positive bacteria atmospheres, due to proteolytic metabolism. Similarly, in the other Gram-negative bacteria studied (*Pseudomonas* spp), counts remained below 6.0 log cfu g^−1^ at the end of storage. Lactic bacteria showed counts close to or above this limit at the end of the study. On the other hand, high oxygen atmosphere conditions will generally stimulate the growth of aerobic bacteria (*Pseudomonas* spp.), which are responsible for bad odors due to the production of NH_3_. In fact, although *Pseudomonas* spp. mainly occur during air storage of raw meat, this microorganism can play a significant role in the spoilage of meat [47]. Regarding the bacteriostatic effect of CO_2_, it is well established that the inhibitory effect of CO_2_ affects Gram-positive bacteria (lactic acid bacteria and *Brochothrix* spp.) to a lesser extent than Gram-negative bacteria (*Pseudomonas* spp. and Enterobacteriaceae) [51]. Concerning differences between dietary groups, statistically significant differences were only observed for lactic acid bacteria, (*p* = 0.063), *Pseudomonas* spp. (*p* = 0.005) and *Brochothrix thermosphacta* (*p* = 0.012) at D16, when the G4 group showed lower counts. This would suggest better preservation of meat in animals that had received the natural compound in the diet. Buchanan [52] established 7 log10 cfu/g as a limit for microbial spoilage of meat and is frequently correlated with sensory deterioration, like off-odors and the presence of slime on the surface of meat [48]. In this study, aerobic bacteria reached levels close to 7 log10 cfu/g on D21; however, sensory deterioration was established before that time. This fact has also been noted by previous authors [53,54,55], who reported that the spoilage can be detected, because of the odor in most foods with more than 6 log10 CFU/g.

No differences were observed in the total bacterial count depending on the pig diet at the end of the experiment. However, TVC was lower in G4 at D0 (*p* = 0.006). In accordance with those results, previous authors [17,19,56] have observed an increase in microbial reductions when organic acids were added to the meat. The highest value for Enterobacteriaceae at D0 was shown in the G4 group. Otherwise, Fabricio et al. [57] did not observe reductions in coliforms and Enterobacteriaceae when spraying poultry carcasses with acetic acid.

The shelf life of meat products at refrigeration is based largely on sensory characteristics and correlates with appearance, color and odor that can be perceived by the consumer [47]. In this study, the effect of dietary adaptation on the sensory parameters evaluated was hardly noticeable. It was only at D16 that the presence of off-odor was higher in groups including the two forms of oleic acid. Nevertheless, taking into account all the parameters evaluated by the panel of testers, values greater than 3 were reached from day 16 of packaging, thus establishing the shelf life of the meat as 11 days. In any case, the shelf life of the Iberian pig meat observed in this study was greater than that shown by previous authors using the same composition of atmosphere [58,59]. The average spoilage incidence time in red meat products was slightly lower and estimated at 9.2 days [47]. In a review of studies published by Luong et al. [47], it was shown that spoilage occurred between the third and ninth day in most cases. In only 13% of cases, the meat reached 16 days before spoilage. As indicated above, the microbiological counts did not reach rejection levels until 21 days, so the limiting factor of the shelf life has been the presence of discoloration and abnormal odors as detected by the panel. It has already been known that there is a fundamental difference between shelf life based on microbial criteria or sensory evaluation. Our results are consistent with Liu et al. [54], studying various atmospheres and storage conditions, and, in most of them, sensory-based shelf life is shorter than microbial shelf life. The development of unpleasant odors is a consequence of the accumulation of lipid and protein oxidation products, as well as derivatives of microbial metabolism [44,53,54]. This data is interesting if we consider that G4 included a natural organic–acid mix, so it was expected that this group would present the consequences of oxidation later. It can be presumed that the effect of the incorporation of oleic acid in different forms increasing the percentage of polyunsaturated fatty acids, which are more susceptible to oxidation, has prevailed over the natural organic-acid mix.

## 5. Conclusions

The inclusion of oleic acid in its solid form seems to have a better level of assimilation by the Iberian pig. This fact is reflected in the variations in meat quality (in terms of fat, fatty acid profile and sensory characteristics). The effect of organic-acid mix on the shelf life of the meat appears to be limited. Meat from the G4 group showed a lower value of TVC at D0, but did not increase shelf life at the end of the experiment. New studies are required to verify the effect of the organic-acid mix.

## Figures and Tables

**Table 1:** Nutritive value and fatty acid composition of different fats.

	Pig Fat	Solid Oleic Acid	Oleic High Sunflower Oil
Metabolizable Energy (kcal/kg)	8288.00	6640.00	8100.00
Crude fat (%)	99.00	83.00	95.10
Fatty acid profile (g/100 g of fat)			
C14:0	1.30	0.25	nd
C16:0	23.80	10.50	6.40
C18:0	13.50	3.50	5.00
C18:1 *n-9*	41.20	68.50	22.60
C18:2 *n-6*	10.20	8.00	63.00
C18:3 *n-3*	1.00	0.70	0.50

nd: not detected.

**Table 2:** Composition and nutrient contents of experimental diets.

	Growing 2				Finishing			
	G1	G2	G3	G4	G1	G2	G3	G4
**Ingredients (g/100 g)**								
Barley	50.00	50.00	50.00	50.00	48.21	45.60	46.94	46.94
Wheat	30.65	30.54	30.65	30.61	35.00	36.06	35.00	35.08
Soybean meal	12.49	12.48	12.50	12.48	8.23	8.59	8.54	8.54
Wheat bran	2.50	2.50	2.50	2.50	3.00	3.00	3.00	3.00
Calcium carbonate	1.66	1.55	1.55	1.55	1.48	1.44	1.82	1.70
Monocalcium Phosphate	0.58	0.58	0.58	0.58	0.45	0.45	0.45	0.45
Common Salt	0.50	0.50	0.50	0.50	0.50	0.50	0.50	0.50
Premix ^1^	0.30	0.30	0.30	0.30	0.30	0.30	0.30	0.30
Pig Fat	1.20	0.00	0.00	0.00	2.61			
Lysine	0.20	0.20	0.20	0.20	0.21	0.21	0.21	0.21
Oleic high sunflower oil			0.62	0.62			1.73	1.73
Oleic Solid Acid		1.35	0.60	0.60		3.85	1.50	1.50
Organic-acid mix				0.05				0.05
Total	100.00	100.00	100.00	100.00	100.00	100.00	100.00	100.00
**Chemical composition (g/1000 g)**								
Metabolizable Energy (kcal/kg)	3153	3149	3153	3151	3250	3250	3250	3250
Crude protein	14.50	14.50	14.50	14.50	13.00	13.00	13.00	13.00
Crude Fiber	4.40	4.40	4.41	4.41	4.26	4.18	4.22	4.22
Crude Fat	3.00	3.00	3.00	3.00	4.50	5.05	4.84	4.84
Ash (550 °C)	5.52	5.44	5.49	5.48	5.03	5.43	5.54	5.42
Starch	45.43	45.45	45.43	45.37	47.18	46.43	46.51	46.51
C16:0	0.58	0.43	0.39	0.39	0.94	0.64	0.52	0.52
C18:0	0.18	0.07	0.06	0.06	0.37	0.16	0.12	0.12
C18:1 *n-9*	0.70	1.02	1.08	1.08	1.34	2.50	2.50	2.50
C18:2 *n-6*	0.87	0.89	0.89	0.89	0.99	1.09	1.06	1.06
Calcium	0.95	0.95	0.95	0.95	0.85	1.10	1.09	1.04
Total Phosphorus	0.55	0.55	0.55	0.55	0.50	0.50	0.50	0.50
Methionine	0.22	0.22	0.22	0.22	0.20	0.20	0.20	0.20
M + C	0.52	0.52	0.52	0.52	0.48	0.48	0.48	0.48
Lysine	0.80	0.80	0.80	0.80	0.70	0.70	0.70	0.70
Tryptophan	0.19	0.19	0.19	0.19	0.17	0.17	0.17	0.17
Threonine	0.50	0.50	0.50	0.50	0.44	0.44	0.44	0.44
Sodium	0.22	0.22	0.22	0.22	0.22	0.22	0.22	0.22

Premix ^1^ (Arganda del Rey, Madrid, Spain). Values given (mg) per kg of feed: E5 manganese (manganese oxide)-30.0; iodine-1.0; E6 zinc (zinc oxide)-100.0; E4 copper (cupric sulfate pentahydrate)-10.0; E8 selenium (sodium selenite)-0.2; E1 iron (ferrous carbonate)-100.0; vitamin E-30.0; vitamin K3-1.0; vitamin B1-1.0; vitamin B2-2.0; vitamin B6-1.0; vitamin B12-0.06. Values given (IU) per kg of feed: vitamin A-6500.0; vitamin D3-1500.0, 6-phytases EC 3.1.3.26 (FTU) 500, endo-1,3 (4) -B-Glucanase EC 3.2.1.6 (U/Kg)-152, Endo-1,4-B-Xylanase EC3.2.1.8(U/Kg)-1220.

**Table 3:** Live and carcass weight and back-fat thickness (mean ± standard deviation) of pigs from dietary experimental groups.

Slaughter Analysis	G1	G2	G3	G4	*p*
Live weight (kg)	166.8 ± 11.56	165.04 ± 12.80	163.98 ± 17.80	162.84 ± 13.73	0.671
Carcass weight (kg)	75.25 ± 7.57	75.88 ± 5.30	76.14 ± 3.99	79.31 ± 6.52	0.365
Thickness of backfat (6th rib) (cm)	7.29 ± 1.14	7.25 ± 1.0	6.45 ± 1.11	6.5 ± 0.91	0.112
Thickness of back fat (3rd lumbar vertebra)	8.88 ^a^ ± 1.00	7.50 ^b^ ± 0.86	7.81 ^b^ ± 0.78	8.31 ^a,b^ ± 0.95	0.030

G1—control (pig fat); G2—solid oleic acid; G3—oleic high sunflower oil + solid oleic acid; and G4—oleic high sunflower oil + solid oleic + natural organic-acid mix. Results are presented as mean ± standard deviation. ^a,b^: different superscripts indicate significant differences within a row (*p* < 0.05).

**Table 4:** Fatty acid profile (mean ± standard deviation) of subcutaneous fat from dietary experimental groups.

	G1	G2	G3	G4	*p*
C12:0	0.08 ± 0.08	0.08 ± 0.01	0.08 ± 0.10	0.08 ± 0.01	0.413
C14:0	1.44 ± 0.11	1.395 ± 0.07	1.43 ± 0.13	1.36 ± 0.12	0.615
C16:0	24.81 ± 0.77	24.86 ± 0.19	25.44 ± 0.80	24.87 ± 0.49	0.266
C16:1 *n-7*	2.43 ± 0.26	2.53 ± 0.21	2.20 ± 0.21	2.21 ± 0.39	0.142
C17:0	0.39 ± 0.07	0.33 ± 0.10	0.36 ± 0.08	0.29 ± 0.08	0.202
C17:1 *n-6*	0.40 ± 0.09	0.34 ± 0.10	0.33 ± 0.08	0.27 ± 0.07	0.113
C18:0	12.99 ^a,b^ ± 0.75	12.37 ^b^ ± 0.92	14.13 ^a^ ± 0.97	13.71 ^a^ ± 1.54	0.049
C18:1 *n-9*	47.47 ^a,b^ ± 0.83	48.65 ^a^ ± 1.12	46.25 ^b^ ± 1.12	47.79 ^b^ ± 1.41	0.013
C18:2 *n-6*	8.17 ± 0.67	7.68 ± 0.56	7.96 ± 0.71	7.57 ± 0.58	0.362
C18:3 *n-3*	0.45 ± 0.05	0.42 ± 0.05	0.425 ± 0.05	0.39 ± 0.04	0.244
C20:0	0.18 ± 0.02	0.21 ± 0.02	0.20 ± 0.02	0.22 ± 0.03	0.107
C20:1 *n-9*	1.18 ± 0.20	1.13 ± 0.11	1.18 ± 0.10	1.23 ± 0.18	0.743
SFA	39.89 ^b^ ± 1.39	39.23 ^b^ ± 0.94	41.65 ^a^ ± 1.24	40.53 ^a,b^ ± 1.63	0.031
MUFA	51.48 ^a,b^ ± 0.90	52.65 ^a^ ± 1.46	49.97 ^b^ ± 1.19	51.50 ^a,b^ ± 1.80	0.026
PUFA	8.62 ± 0.71	8.11 ± 0.60	8.38 ± 0.76	7.96 ± 0.61	0.358
PUFA/SFA	0.22 ± 0.02	0.21 ± 0.01	0.20 ± 0.21	0.20 ± 0.02	0.333
*n-6*/*n-3*	17.33 ^a^ ± 0.67	17.23 ^a^ ± 0.56	18.89 ^a,b^ ± 0.70	20.00 ^b^ ± 0.00	0.010

G1: control (pig fat); G2: solid oleic acid; G3: oleic high sunflower oil + solid oleic acid; and G4: oleic high sunflower oil + solid oleic + natural organic-acid mix. Results are presented as mean ± standard deviation. ^a,b^: different superscripts indicate significant differences within a row (*p* < 0.05).

**Table 5:** Fatty acid profile (mean ± standard deviation) of m. longissimus thoracis from dietary experimental groups.

	G1	G2	G3	G4	*p*
C12:0	0.14 ^a^ ± 0.047	0.08 ^b^ ± 0.01	0.09 ^b^ ± 0.01	0.09 ^b^ ± 0.03	0.023
C14:0	1.51 ± 0.09	1.45 ± 0.11	1.46 ± 0.09	1.49 ± 0.01	0.618
C14:1 *n-5*	0.03 ^b^ ± 0.01	0.03 ^b^ ± 0.00	0.03 ^a^ ± 0.00	0.04 ^a^ ± 0.01	0.000
C15:0	0.02 ± 0.00	0.02 ± 0.00	0.03 ± 0.01	0.02 ± 0.01	0.253
C16:0	25.76 ± 1.73	25.86 ± 0.08	25.51 ± 0.79	25.75 ± 0.58	0.889
C16:1 *n-7*	4.16 ± 0.98	3.97 ± 0.32	4.16 ± 0.38	3.99 ± 0.34	0.836
C17:0	0.14 ± 0.022	0.15 ± 0.032	0.16 ± 0.04	0.14 ± 0.013	0.466
C18:0	12.47 ± 1.60	12.29 ± 0.69	11.62 ± 0.47	12.35 ± 0.57	0.199
C18:1 *n-7*	3.87 ± 0.72	3.85 ± 0.24	4.08 ± 0.17	3.85 ± 0.26	0.529
C18:1 *n-9*	45.82 ± 1.68	46.59 ± 0.89	46.40 ± 1.18	45.85 ± 0.96	0.396
C18:2 t10-c12	0.09 ^a^ ± 0.012	0.09 ^a^ ± 0.01	0.08 ^a^ ± 0.01	0.08 ^b^ ± 0.01	0.074
C18:2 *n-6*	3.46 ± 0.61	3.09 ± 0.31	3.62 ± 0.76	3.63 ± 0.63	0.175
C18:3 *n-3*	0.15 ± 0.026	0.13 ± 0.011	0.14 ± 0.022	0.15 ± 0.025	0.176
C18:3 c9-c11-c13	0.01 ± 0.00	0.01 ± 0.00	0.02 ± 0.00	0.13 ± 0.00	0.249
C18:3 *n-6*	0.04 ± 0.01	0.04 ± 0.01	0.04 ± 0.01	0.04 ± 0.01	0.140
C20:0	0.18 ± 0.02	0.17 ± 0.02	0.18 ± 0.01	0.19 ± 0.02	0.309
C20:1 *n-9*	0.93 ± 0.06	0.97 ± 0.09	0.97 ± 0.07	0.93 ± 0.08	0.564
C20:2 *n-6*	0.18 ± 0.02	0.17 ± 0.02	0.19 ± 0.03	0.19 ± 0.02	0.323
C20:3 *n-6*	0.08 ± 0.02	0.07 ± 0.01	0.09 ± 0.02	0.02 ± 0.02	0.103
C20:4 *n-3*	0.02 ± 0.00	0.02 ± 0.00	0.20 ± 0.00	0.02 ± 0.00	0.3628
C20:4 *n-6*	0.46 ^a,b^ ± 0.18	0.43 ^b^ ± 0.11	0.58 ^a,b^ ± 0.22	0.61 ^a^ ± 0.19	0.088
C20:5 *n-3*	0.02 ± 0.01	0.02 ± 0.00	0.02 ± 0.00	0.02 ± 0.01	0.448
C21:0	0.03 ± 0.00	0.03 ± 0.00	0.03 ± 0.00	0.03 ± 0.00	0.413
C22:1 *n-9*	0.01 ^b^ ± 0.00	0.01 ^b^ ± 0.00	0.02 ^a^ ± 0.00	0.02 ^a^ ± 0.00	0.004
C22:4 *n-6*	0.08 ^a,b^ ± 0.02	0.07 ^b^ ± 0.01	0.10 ^a^ ± 0.03	0.10 ^a^ ± 0.0229	0.024
C22:5 *n-3*	0.06 ± 0.02	0.05 ± 0.01	0.07 ± 0.02	0.07 ± 0.018	0.119
C22:6 *n-3*	0.02 ^b^ ± 0.00	0.01 ^b^ ± 0.00	0.02 ^a^ ± 0.01	0.02 ^a^ ± 0.01	0.026
SFA	40.31 ± 3.19	40.16 ± 1.45	39.15 ± 1.54	40.12 ± 1.00	0.516
MUFA	54.98 ± 3.24	55.63 ± 1.04	55.85 ± 1.28	54.86 ± 1.07	0.587
PUFA	4.67 ± 0.92	4.20 ± 0.47	4.99 ± 1.12	5.01 ± 0.89	0.157
*n-6*/ *n-3*	15.35 ^b^ ± 0.38	15.49 ^b^ ± 0.45	16.03 ^a^ ± 0.62	16.49 ^a^ ± 0.43	0.000

G1: control (pig fat); G2: solid oleic acid; G3: oleic high sunflower oil + solid oleic acid; and G4: oleic high sunflower oil + solid oleic + natural organic-acid mix. Results are presented as mean ± standard deviation. ^a,b^: different superscripts indicate significant differences within a row (*p* < 0.05).

**Table 6:** Proximate composition and instrumental quality parameters (mean ± standard deviation) of longissimus thoracis muscle from dietary experimental groups.

	G1	G2	G3	G4	*p*
Moisture (%)	66.75 ± 2.51	66.57 ± 1.77	67.81 ± 2.41	68.63 ± 2.25	0.159
Fat (%)	11.53 ^a^ ± 2.77	10.84 ^a^ ± 2.28	9.66 ^a,b^ ± 3.08	8.14 b ± 2.19	0.047
Protein (%)	20.51 ± 0.73	20.83 ± 0.6	20.92 ± 0.76	21.01 ± 0.93	0.498
Lightness (L*)	69.59 ± 3.46	69.86 ± 2.12	69.46 ± 1.62	69.96 ± 1.17	0.96
Redness index (a*)	6.19 ± 0.73	6.71 ± 1.05	5.94 ± 0.54	6.12 ± 0.8	0.183
Yellowness index (b*)	8.92 ± 1.32	9.22 ± 0.73	8.65 ± 1.09	8.96 ± 1.22	0.546
pH	5.74 ± 0.04	5.73 ± 0.039	5.71 ± 0.054	5.75 ± 0.04	0.307
Cooking losses (%)	15.90 ^a,b^ ± 1.9	14.23 b ± 3.45	15.87 ^a,b^ ± 2.11	18.84 ^a^ ± 2.56	0.003
Warner–Bratzler shear force (kg)	3.2 ± 1.08	3.23 ± 0.98	3.65 ± 0.65	3.89 ± 1.13	0.333

G1: control (pig fat); G2: solid oleic acid; G3: oleic high sunflower oil + solid oleic acid; and G4: oleic high sunflower oil + solid oleic + natural organic-acid mix. Results are presented as mean ± standard deviation. ^a,b^: different superscripts indicate significant differences within a row (*p* < 0.05).

**Table 7:** Sensorial parameters (mean ± standard deviation) of meat from dietary experimental groups.

	G1	G2	G3	G4	*p*
External appreciation of fresh meat					
Color	2.80 ± 0.71	2.83 ± 0.71	2.83 ± 0.82	2.95 ± 0.83	0.925
Marbling	3.75 ^a,b^ ± 0.66	4.28 ^a^ ± 0.57	4.00 ^a,b^ ± 0.63	3.30 ^b^ ± 0.81	0.002
Fat color	1.07 ± 0.0.24	1.00 ± 0.00	1.00 ± 0.00	1.00 ± 0.00	0.293
Fat distribution	3.95 ^a,b^ ± 0.68	4.23 ^a^ ± 0.69	4.16 ^a^ ± 0.40	3.50 ^b^ ± 0.83	0.010
Characteristics of cooked meat					
Color	1.77 ± 0.69	1.66 ± 0.48	2.25 ± 0.61	11.87 ± 0.60	0.228
Odor Intensity	3.12 ± 0.60	3.11 ± 0.76	3.50 ± 0.54	3.98 ± 0.82	0.476
Tenderness	3.77 ^a^ ± 0.69	3.67 ^a^ ± 1.02	3.17 ^a,b^ ± 0.41	3.12 ^b^ ± 0.65	0.036
Chewiness	2.85 ^a^ ± 0.81	2.66 ^a^ ± 0.77	2.83 ^a,b^ ± 0.41	2.43 ^b^ ± 0.67	0.014
Juiciness	3.52 ^a^ ± 0.50	3.50 ^a,b^ ± 0.56	3.03 ^b^ ± 0.63	2.95 ^b^ ± 0.60	0.002
Flavor intensity	3.45 ± 0.51	3.44 ± 0.51	3.333 ± 0.52	3.37 ± 0.66	0.949
Flavor quality	3.65 ± 0.67	3.78 ± 0.55	3.33 ± 0.52	3.35 ± 0.58	0.117
Overall liking	3.53 ^a,b^ ± 0.749	3.72 ^a^ ± 0.46	3.17 ^a,b^ ± 0.41	3.05 ^b^ ± 0.39	0.016

G1: control (pig fat); G2: solid oleic acid; G3: oleic high sunflower oil + solid oleic acid; and G4: oleic high sunflower oil + solid oleic + natural organic-acid mix. Results are presented as mean ± standard deviation. ^a,b^: different superscripts indicate significant differences within a row (*p* < 0.05).

**Table 8:** Microbial counts of pig meat from dietary experimental groups packaged in a modified atmosphere throughout the storage (0, 4, 8, 11, 16 and 21 days) in commercial display conditions.

Microbial Counts (Log CFU/g)	Pig Diet	D0	D4	D8	D11	D16	D21	Days *p*-Value
Total viable counts (TVC)	G1	3.9 ^a,A^	3.9 ^a,A^	4.2 ^a,b,A^	4.4 ^A^	6.2 ^B^	7.4 ^B^	0.000
	G2	3.3 ^b,A^	2.9 ^b,A^	3.5 ^A,b^	5.2 ^B^	5.0 ^B^	7.0 ^C^	0.000
	G3	4.1 ^a,A^	3.4 ^a,b,A^	4.6 ^a,B^	4.7 ^B^	6.3 ^C^	7.3 ^D^	0.000
	G4	3.4 ^b,A^	3.5 ^a,b,A^	3.4 ^b,A^	4.8 ^B^	5.7 ^B^	6.9 ^C^	0.000
	Pig diet *p*-value	0.006	0.075	0.074	0.288	0.254	0.721	
*Enterobacteria*	G1	1.0 ^a,A^	1.4 ^A^	1.7 ^A,B^	3.0 ^B^	3.5 ^B^	5.8 ^a,b,C^	0.000
	G2	1.0 ^a,A^	1.6 ^A^	2.0 ^A,B^	3.0 ^B^	3.8 ^B^	5.2 ^b,C^	0.000
	G3	1.0 ^a,A^	1.4 ^A^	1.7 ^A^	3.0 ^B^	4.4 ^B^	5.1 ^b,C^	0.000
	G4	1.5 ^b,A^	1.6 ^b,A^	1.7 ^A^	3.0 ^B^	4.0 ^B^	6.0 ^a,C^	0.002
	Pig diet *p*-value	0.063	0.596	0.916	0.839	0.921	0.086	
Lactic acid bacteria (LAB)	G1	1.7 ^A^	2.4 ^A,B^	2.7 ^AB^	3.2 ^B^	5.7 ^a,^^C^	6.4 ^C^	0.000
	G2	1.9 ^A^	1.8 ^A^	2.6 ^A,B^	3.4 ^B^	3.8 ^b,B^	5.4 ^C^	0.000
	G3	2.0 ^A^	2.0 ^A^	3.2 ^B^	4.2 ^B^2	5.6 ^a,B,C^	6.4 ^C^	0.000
	G4	2.0 ^A^	2.0 ^A^	2.3 ^A^	4.1 ^B^	4.5 ^b,B^	5.9 ^C^	0.000
	Pig diet *p*-value	0.665	0.438	0.630	0.254	0.063	0.129	
*Pseudomonas* spp.	G1	2.0 ^A^	2.2 ^A^	3.0 ^a,B^	3.4 ^a,B^	4.4 ^a,B,C^	6.5 ^C^	0.000
	G2	2.0 ^A^	2.0 ^A^	2.0 ^b,A^	2.0 ^b,A^	2.6 ^b,A,B^	6.4 ^B^	0.000
	G3	2.0 ^A^	2.0 ^A^	3.2 ^a,B^	3.9 ^a,A,B^	4.7 ^a,B^	6.0 ^C^	0.000
	G4	2.2 ^A^	2.2 ^A^	2.0 ^b,A^	2.9 ^b,A^	2.0 ^b,A^	6.4 ^B^	0.000
	Pig diet *p*-value	0.441	0.596	0.007	0.096	0.005	0.559	
*Brochothrix thermosphacta*	G1	2.2 ^A^	2.2 ^A^	2.9 ^A,B^	4.1 ^A,B^	4.8 ^a,b,B^	5.5 ^B^	0.003
	G2	2.0 ^A^	2.0 ^A^	2.5 ^A^	2.4 ^A^	2.0 ^b,A^	4.2 ^B^	0.002
	G3	2.0 ^A^	2.0 ^A^	2.5 ^A,B^	3.6 ^B^	5.7 ^a,C^	5.7 ^C^	0.000
	G4	2.0	2.0	2.0	2.2	2.9 ^b^	3.7	0.449
	Pig diet *p*-value	0.441	0.441	0.223	0.111	0.012	0.449	

G1: control (pig fat); G2: solid oleic acid; G3: oleic high sunflower oil + solid oleic acid; and G4: oleic high sunflower oil + solid oleic + natural organic-acid mix. Results are presented as mean ± standard deviation. ^A,B,C,D^ Averages for each microbial group with a different capital letter mean significant differences (*p* < 0.05) between days of storage within diets. ^a,b^: Averages for each microbial group with a different lower-case letter mean significant differences (*p* < 0.05) between diets within days of storage.

## Data Availability

Data are not available in public datasets, please contact the authors.
